# Hypoxia Promotes a Mixed Inflammatory-Fibrotic Macrophages Phenotype in Active Sarcoidosis

**DOI:** 10.3389/fimmu.2021.719009

**Published:** 2021-08-11

**Authors:** Florence Jeny, Jean-François Bernaudin, Dominique Valeyre, Marianne Kambouchner, Marina Pretolani, Hilario Nunes, Carole Planès, Valérie Besnard

**Affiliations:** ^1^INSERM UMR 1272, Sorbonne Paris-Nord University, Bobigny, France; ^2^AP-HP, Pulmonology Department, Avicenne Hospital, Bobigny, France; ^3^Faculty of Medicine, Sorbonne University, Paris, France; ^4^AP-HP, Pathology Department, Avicenne Hospital, Bobigny, France; ^5^Inserm UMR1152, Physiopathology and Epidemiology of Respiratory Diseases, Paris, France; ^6^Faculty of Medicine, Bichat Hospital, Paris University, Paris, France; ^7^Laboratory of Excellence, INFLAMEX, Paris University, DHU FIRE, Paris, France; ^8^AP-HP, Physiology Department, Avicenne Hospital, Bobigny, France

**Keywords:** pulmonary sarcoidosis, hypoxia-inducible factor 1, plasminogen activator inhibitor-1, macrophages, fibrosis, monocytes

## Abstract

**Background:**

Macrophages are pivotal cells in sarcoidosis. Monocytes-derived (MD) macrophages have recently been demonstrated to play a major role especially in pulmonary sarcoidosis. From inflammatory tissues to granulomas, they may be exposed to low oxygen tension environments. As hypoxia impact on sarcoidosis immune cells has never been addressed, we designed the present study to investigate MD-macrophages from sarcoidosis patients in this context. We hypothesized that hypoxia may induce functional changes on MD-macrophages which could have a potential impact on the course of sarcoidosis.

**Methods:**

We studied MD-macrophages, from high active sarcoidosis (AS) (n=26), low active or inactive sarcoidosis (IS) (n=24) and healthy controls (n=34) exposed 24 hours to normoxia (21% O_2_) or hypoxia (1.5% O_2_). Different macrophage functions were explored: hypoxia-inducible factor-1α (HIF-1α) and nuclear factor-kappa B (NF-κB) activation, cytokines secretion, phagocytosis, CD80/CD86/HLA-DR expression, profibrotic response.

**Results:**

We observed that hypoxia, with a significantly more pronounced effect in AS compared with controls and IS, increased the HIF-1α trans-activity, promoted a proinflammatory response (TNFα, IL1ß) without activating NF-κB pathway and a profibrotic response (TGFß1, PDGF-BB) with PAI-1 secretion associated with human lung fibroblast migration inhibition. These results were confirmed by immunodetection of HIF-1α and PAI-1 in granulomas observed in pulmonary biopsies from patients with sarcoidosis. Hypoxia also decreased the expression of CD80/CD86 and HLA-DR on MD-macrophages in the three groups while it did not impair phagocytosis and the expression of CD36 expression on cells in AS and IS at variance with controls.

**Conclusions:**

Hypoxia had a significant impact on MD-macrophages from sarcoidosis patients, with the strongest effect seen in patients with high active disease. Therefore, hypoxia could play a significant role in sarcoidosis pathogenesis by increasing the macrophage proinflammatory response, maintaining phagocytosis and reducing antigen presentation, leading to a deficient T cell response. In addition, hypoxia could favor fibrosis by promoting profibrotic cytokines response and by sequestering fibroblasts in the vicinity of granulomas.

## Introduction

Sarcoidosis is a systemic granulomatous disease of unknown cause affecting the lung with a prevalence close to 90% (1, 2). Granulomas, the key lesion in sarcoidosis, are well circumscribed and coalescent clusters of macrophage-derived epithelioid and giant cells, associated with lymphocytes, subsequently wrapped by lamellar fibrosis (3). The prevailing hypothesis explaining granuloma formation is that exposure to unknown antigens triggers a disproportionate inflammatory response in genetically predisposed individuals (4). Sarcoidosis is particularly challenging in regards of identifying factors involved in initiation of inflammation, granuloma perpetuation and evolution towards fibrosis as observed mostly in pulmonary, but also in hepatic, renal or cardiac involvement (3, 5–7). These events are determinant for sarcoidosis outcomes which vary from benign to life-threatening especially due to pulmonary fibrosis and/or pulmonary hypertension (8).

Macrophages are pivotal cells in the sequence of events associated with sarcoidosis pathogenesis, granulomas having been clearly demonstrated constituted of macrophages-derived cells (9–13). Different types of macrophage populations (alveolar and interstitial) derivating from distinct precursors of embryonic/fetal and myeloid origin, reside in the lung (14, 15). Notably, upon injury, the myeloid precursors generate monocyte-derived (MD) macrophages, though to play a major role in fibrosis (16) and sarcoidosis (11, 17–20).

In the lung of patients, these cells are an important source of TNFα, a major cytokine in the pathogenesis of sarcoidosis (11). Circulating mononuclear cells from sarcoidosis patients are also able to reconstitute granulomas *in vitro*, and present a specific transcriptional profile compared to other granulomatosis such as tuberculosis (17, 18). In addition, analysis of granulomas from sarcoidosis recurrence after lung transplantation demonstrated that constitutive cells derived from recipient blood cells (19, 20), indicating that peripheral blood cells are major contributors in granuloma formation.

Hypoxia, defined as an imbalance between impaired tissue oxygen supply and cell demand, is a micro-environmental condition known to modulate innate and adaptive immunity (21) by promoting pro-inflammatory response (22) and to contribute to the pathophysiology of fibrotic diseases (23). Hypoxia impact on macrophages is particularly relevant to study in sarcoidosis as a previous study showed that the absence of in-depth vascularization makes sarcoidosis granulomas hypoxic (24) as for tuberculous granulomas (25). Moreover, from inflammatory tissues to granulomas, MD-macrophages may be exposed to low oxygen tension environments mainly due to O_2_ consumption related to hypermetabolism of inflammatory cells (26). The cellular response to hypoxia is mostly controlled by the hypoxia-inducible factor (HIF) transcription factor and its target genes harboring the hypoxia-response element (27). Several studies in sarcoidosis reported the expression of HIF-target genes within lung and lymph node granulomas, while HIF-1α expression was inconsistently found (28, 29). Hypoxia and the HIF-1 signaling impact on the processes controlling granuloma evolution are still unclear and their effects on sarcoidosis immune cells have never been addressed.

We hypothesized that hypoxia may induce macrophage functional changes with a potential impact on the course of the disease. Therefore, we studied the consequences of hypoxia on a set of main functions of MD-macrophages from sarcoidosis patients and healthy controls. We show that hypoxia had a significant impact on MD-macrophages from sarcoidosis patients, with the strongest effect seen in patients with high active disease. Therefore, hypoxia could play a significant role in sarcoidosis pathogenesis and fibrosis outcome.

## Materials and Methods

Additional details are provided in the **Online Supplement**.

### Patients and Controls

This prospective monocentric study was conducted in the Pulmonary Department of the Avicenne University Hospital, France between 2017 and 2019 and received institutional review board approval (CPP Ile-de-France X 2016-10-02) according to French legislation. Written informed consent for all participants was obtained for biological investigations.

Patients were over 18 with a pulmonary sarcoidosis according to guidelines (30, 31). The diagnosis of sarcoidosis was validated in multidisciplinary meetings with histopathology confirmation in 46 of the 50 patients, and 4 patients had an initial Lofgren syndrome.

Individuals with corticosteroid/immunosuppressive therapy in the past 6 months were excluded. As the lung is the most affected organ in sarcoidosis and disease activity is difficult to define, we used a computed tomography (CT) score developed in pulmonary sarcoidosis: the *abbreviated Computed-Tomography Activity Score* (aCTAS) (32). Briefly, aCTAS comprised the sum of the presence (1) or absence (0) of nodularity, ground glass opacification, interlobular septal thickening and consolidation (see **Figure E1**). According to aCTAS, patients were divided in high Active Sarcoidosis (AS) (n=26) with an aCTAS≥2, and low active or Inactive Sarcoidosis (IS) groups (n=24) with an aCTAS<2. Bioclinical and imaging data were recorded at the time of blood draw. Healthy volunteers (n=34) over 18, with no history of sarcoidosis or any known current disease were evaluated as “controls” and matched for age (± 5 years), sex and smoking status with sarcoidosis patients.

In addition, paraffin embedded surgical pulmonary biopsies done before this study for diagnosis in three patients with active sarcoidosis were retrieved from the pathology department archives.

### Peripheral Blood Mononuclear Cells (PBMCs) Isolation and Macrophage Differentiation

Twenty mL of total blood sampled in EDTA tubes for routine hematology analysis were collected. We isolated PBMCs using Ficoll density gradient separation and monocytes were purified using the Pan Monocyte Isolation Kit (Miltenyi-Biotec, France). A >90% purity of enriched monocytes was controlled by flow cytometry using APC-CyTM7 mouse anti-Human CD14 (BD Biosciences #557831). Monocytes were cultured in RPMI containing 10% fetal bovine serum (FBS) and differentiated into macrophages with M-CSF (33) (Bio-Techne, France) at physiologic circulating concentration of 5ng/ml (34) for 10 days at 21% O_2_. The yield of isolated monocytes from either controls or sarcoidosis patients (AS and IS) allowed to only perform two or three different types of assays on the same sample.

#### Normoxic and Hypoxic Culture Conditions

After these 10 days of differentiation, MD-macrophages were maintained under either hypoxic or normoxic conditions for 24hrs. Cell cultures under hypoxic conditions were performed in a hermetic chamber containing 1.5% O_2_ for 24hrs to reproduce deep hypoxia (pO_2_<10mmHg) already evidenced *in vivo* in sarcoid granulomas by fluoromisonidazole uptake (35). Normoxic cells were maintained at 21% O_2_ for 24hrs as used in sarcoidosis *in vitro* models (18, 36). After normoxic or hypoxic exposures, cells or cell lysates and/or supernatants were processed for experiments 2.4 to 2.9.

### HIF-1α and Nuclear Factor-kappa B (NF-κB) Activation Assays

After hypoxia or normoxia for 24hrs, cell extracts from MD-macrophages were obtained by addition of 100µL lysis buffer (250 mM NaCl, 50 mM HEPES pH 7.0, 5 mM EDTA, 1 mM dithiothreitol (DTT), 0.1% Nonidet NP40, 10 μg/ml aprotinin, 10 μg/ml leupeptin, 50 μg/ml phenylmethylsulfonyl fluoride, 2 mM sodium pyrophosphate, 1 mM sodium orthovanadate). After centrifugation (10 000×*g*, 30 min, 4°C), cell lysate protein content was determined using the Pierce BCA Protein Assay Kit (Thermo Fisher Scientific, France #23225). We evaluated the activation status of HIF-1α, NF-κB-p50 and -p65 using specific oligonucleotide DNA-binding enzyme-linked immunosorbent assays (ELISA) based kit (TransAM^®^ HIF-1 (#47096), NF-kB p50 (#41096) and NF-kB p65 (#40096), Active Motif, Belgium) according to the manufacturer’s protocol.

### Immunofluorescent Staining

We cultured MD-macrophages on Labtek 8 well-chambers slides (Ibidi, France) at a density of 0.2 10^6^ cells/well and placed either under control atmosphere or hypoxia for 24hrs. Slides were incubated with an anti-HIF1α rabbit antibody (HIF1alpha (Novus) # NB100479). To detect an unsuspected hypoxic culture environment, reduction of pimonidazole was assayed. Intracellular hypoxia was assessed by adding 400µM pimonidazole (Hypoxyprobe™) in the medium for 2hrs, then cells were immunostained using an anti-pimonidazole mouse IgG1 monoclonal antibody (MAb1) (1/50 Hypoxyprobe ™ kit). Pimonidazole forms adducts with thiol containing proteins only in cells that have an oxygen concentration less than 14 micromolar – equivalent to a partial pressure pO_2_ = 10 mmHg at 37°C (according to Hypoxyprobe™).

### Phagocytosis Functional Test

Phagocytosis was assayed in MD-macrophages using 1µm fluospheres™ yellow-green (505/515) (#F8823 Invitrogen) with an equivalent of 200 fluospheres per cell (37). MD-macrophages were incubated with fluospheres during 30 min in culture plates and then washed 4 times with PBS to remove free microspheres and fixed with 4% PFA. Phagocytosis was estimated by the number of cells with at least one particle compared to the total number of cells observed on 4 fields (objective x100, 24-well plates).

### Flow Cytometry

Cell surface marker expression of CD14, and/or CD80, CD86, HLA-DR molecules known to be associated with classic macrophage activation (38) and/or CD36, CD163 markers of alternative macrophage activation (38) and/or isotypes-matched IgG were assayed on MD-macrophages using fluorescent labelled antibodies (see **Table E2**). At least 20 x 10^3^ cells per sample were analyzed on a flow cytometer Canto II, BD Biosciences and data analysis was performed using BD FACS Diva™ software. Samples were gated on cells using FSC/SSC and doublet discrimination to identify singlets using SS-W vs SS-A, MD-macrophages were identified on the basis of CD14+ expression (see **Figure E2**).

### RNA Extraction, Reverse Transcription and Quantitative-PCR (RT-qPCR)

The RNA was extracted from MD-macrophages using Trizol (Thermo Fisher Scientific, France #15596018) according to the manufacturer’s protocol. Reverse transcription was performed using reserve transcriptase system from Promega (M-MLV reverse transcriptase #M1708, dNTP mix #U151B, M-MLV RT 5X buffer #M531A Random, RNAsin #N2518).

Messenger RNA levels of cytokines associated with sarcoidosis and fibrosis were assayed by quantitative PCRs in presence of ABsolute QPCR Mix (Thermo Fisher Scientific #AB1162) with primer sets specific to *IL1B, TNF-A, IL-18, IL-10, CXCL8, PDGFB, VEGF, TGFB1* (see **Table E1** *, in the supplement).* A probe set for *UBC* was used as the normalization standard. The PCR and relative quantifications were performed in a real-time PCR system (StepOnePlus Real-Time PCR system, Applied BioSystems).

### Cytokines Assay

“Conditioned media” (CM) from MD-macrophages (500µl of RPMI medium without FBS/1. 10^6^ cells) were collected after 24hrs (under normoxia or hypoxia culture conditions). Presence of cytokines in CM (IL1-ß, CXCL8, TNFα, IL-10, PDGF-BB, PAI-1, VEGF-A, IL-18, IL-5) were assayed (undiluted, in duplicates) using multiplex-bead based assays (ProcartaPlex™ Multiplex Immunoassay (Invitrogen) #PPX-16-MXH497N) according to manufacturer’s instructions. Cytokines to be investigated were chosen according to RTqPCR results, and *Human cytokine antibody array* (proteome profiler™ Bio-techne #ARY022B) (*data not shown; analysis performed on 1 control and 2 active sarcoidosis)*. Cytokine concentrations were automatically calculated or extrapolated if close to the lower limit of detection by the xPONENT software.

Concentrations of active (free) and total (free+latent) TGFß1 in MD-macrophages supernatants were determined by enzyme-linked immunosorbent assays (ELISA) using Duo Set kits (R&D systems #DY240) according to the manufacturer’s instructions.

### Fibroblast Gap-Closure Assays

Migration of normal human lung fibroblasts (NHLFs) was assayed by placing 0.7mm inserts before cell seeding. The percentage of gap-closure was estimated by the difference between the initial and final gap areas at 24hrs of specific culture conditions, relatively to the initial area.

NHLFs were cultured for 24hrs with FBS10% (i) in CM of MD-macrophages in presence of either anti-human plasminogen activator inhibitor-1 (hPAI-1) antibody (Bio-Techne #AF1786) 20ng/ml, or vehicle, or (ii) in RPMI media in presence of either anti-hPAI-1 antibody, or goat IgG isotype control (Bio-Techne # AB-108-C) 20ng/ml, recombinant hPAI-1 (Bio-Techne #1786-PI-010) or vehicle.

### Immunohistochemistry (IHC)

Tissue sections from the three archived pulmonary biopsies were submitted to a microwave antigen-retrieval technique for all antibodies or isotypes. Citrate buffer pH6 was used for PAI-1 and CD68 antibodies and Tris-EDTA pH9 for HIF-1α antibody. Antibodies against: HIF1α (2µg/ml rabbit polyclonal, bs0737, Bioss Inc), PAI-1 (1µg/ml, rabbit polyclonal, Bio-Techne; AF1786) and CD68 [1:1 (ready to use), mouse monoclonal, Dako; IS-61330-2] were used. After being rinsed, tissue sections were incubated with either biotinylated goat anti–rabbit IgG (7.5 μg/ml; Vector Laboratories) or biotinylated horse anti-mouse IgG (7.5 μg/ml; Vector Laboratories) for 30 minutes and detected with an avidin-biotin peroxidase complex detection kit (Vectastain Elite ABC kit; Vector Laboratories) using nickel-diaminobenzidine as a substrate. The precipitation reaction was enhanced with Tris-cobalt, and the sections were counterstained with 0.1% nuclear fast red.

### Statistical Analysis

Demographic results were presented as means ± SD or proportions and compared using one-way Anova, student’s t-test or Chi2 test when appropriate. Biological results were represented using box and whiskers (showing 25^th^ and 75^th^ percentile and median), each point indicates a patient and/or control. Biological results were compared with two-way ANOVA-repeated measures with Sidak *post-hoc* test. PRISM software (v6, GraphPad, USA) was used. A *p* value<0.05 was considered as significant.

## Results

### Patients and Controls

Characteristics and comparison between groups, *i.e.*, AS and IS and controls are shown in **Table 1**. Patients with AS compared to IS had higher angiotensin converting enzyme blood levels (p<0.0001), more impaired forced vital capacity (p=0.006) and diffusing capacity of the lung for carbon monoxide (p=0.003) and had to be more frequently treated for sarcoidosis during the 6 months following inclusion in the study (69% *versus* 21%). The proportion of patients with extra-pulmonary involvement was similar, but Scadding’s radiographic staging was different with a higher proportion of stage 1 in IS.

**Table 1:** Subject characteristics.

Characteristic	Controls (n=34)	AS (n=26)	IS (n=24)	p value
Age, years	44.3 ± 12.5	46.7 ± 12.7	52.3 ± 13.6	0.08
Female (n (%))	17 (50%)	12 (46.6%)	14 (58.3%)	0.7
Current and ex-smoker (n (%))	8 (22.8%)	7 (26.9%)	6 (25%)	0.9
ACE (UI/L) (mean ± SD)	NA	119.3 ± 58	62 ± 30.5	**<0.0001**
Extra thoracic involvement (n (%))	NA	13 (50%)	12 (50%)	1
Scadding CXR stage (n (%))				
0	NA	0 (0%)	4 (16.6%)	**<0.0001**
1		1 (3.8%)	12(50%)	
2		18 (69.2%)	2 (8.3%)	
3		1 (3.8%)	1 (4.1%)	
4		6 (23.1%)	5 (20.8%)	
Pulmonary function				
FVC % [predicted; mean ± SD]	NA	77.6 ± 21.6	94.2 ± 19	**0.006**
DLCO % [predicted; mean ± SD]		58 ± 20.6	74.5 ± 14.2	**0.003**
Treatment initiation* (n (%))	NA	18 (69.2%)	5 (20.8%)	**0.0009**
aCTAS [32] (n (%))	NA			
0		0 (0%)	11 (45.8%)	
1		0 (0%)	13 (54.2%)	
2		18 (69.2%)	0 (0%)	
3		8 (30.7%)	0 (0%)	
4		0 (0%)	0 (0%)	

Data are expressed in mean ± SD or N (%); groups were compared for continuous variables with one-way ANOVA test (3 groups) or t-student test (2 groups) and for categorical variables with Chi^2^ test; *Immunosuppressive or corticosteroid treatment initiation ≤ 6 months after inclusion. AS, high active sarcoidosis, IS, low active or inactive sarcoidosis ACE, angiotensin converting enzyme; CXR, chest x-ray; FVC, forced vital capacity; DLCO, diffusing capacity of the lung for carbon monoxide; aCTAS, abbreviated CT activity Score (32); NA, not applicable.

Bold characters highlight significant values.

### HIF-1α Is Activated by Hypoxia in MD-Macrophages From High Active Sarcoidosis

**Figure 1:**
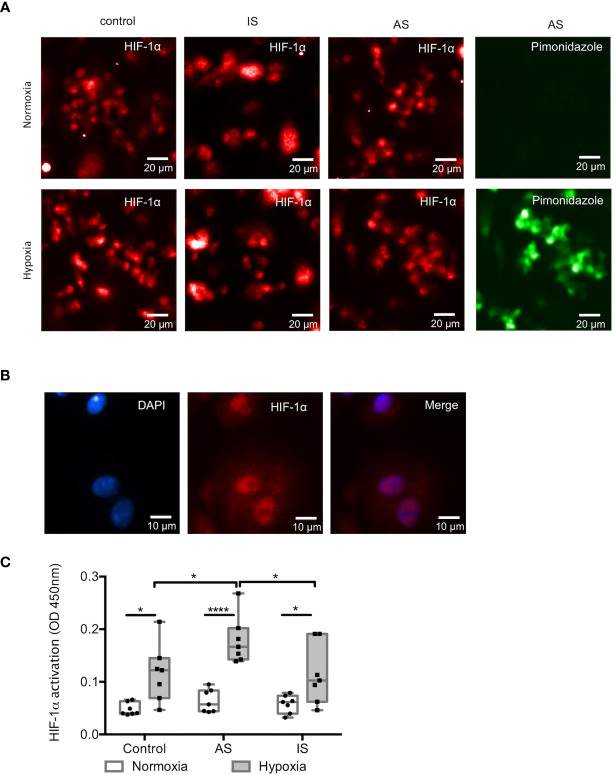
HIF-1α is activated by hypoxia in MD-macrophages from high active sarcoidosis. **(A, B)** Representative immunofluorescence staining of MD-macrophages. **(A)** Similar presence of HIF-1α (in red) in controls and in high active pulmonary sarcoidosis (AS) and low active or inactive sarcoidosis (IS) patients in both normoxia and hypoxia. Pimonidazole staining (in green) of MD-macrophages from AS is absent in normoxia (magnification x200). **(B)** Nuclear staining with DAPI (in blue) and accumulation of HIF-1α (in red) in MD-macrophages. Overlay image showing HIF-1α nuclear localization (magnification x400) (same results were found in 3 patients and 3 controls). **(C)** HIF-1α activation (measured as OD 450nm) assessed by TransAM^®^ HIF-1α in controls, AS and IS patients, after 24hrs of normoxia or hypoxia. Results are expressed as box plot showing 25^th^ and 75^th^ percentile and median, each point indicates a patient and/or control (n= 7/group). *p < 0.05; ****p < 0.0001 in two-way ANOVA-repeated measures with Sidak *post-hoc* test.

The transcription factor HIF-1α was immunodetected in MD-macrophages both in normoxic and hypoxic conditions, similarly between sarcoidosis and control groups (**Figure 1A**) HIF-1α was immunolocalized in the cytoplasm and nucleus of MD-macrophages (**Figure 1B**). Pimonidazole staining was only present in response to hypoxia and absent in normoxia (**Figure 1A**), suggesting that HIF-1α stabilization in MD-macrophages in normoxia is probably induced by other stimuli than hypoxia. This also demonstrates that MD-macrophages exposed to 1.5% oxygen have a partial pressure in oxygen ≤ 10 mmHg. HIF-1α activation status in MD-macrophages was assayed using a specific oligonucleotide-binding test. In normoxia, HIF-1α was similarly activated at a very low level in controls and sarcoidosis groups (**Figure 1C**). In contrast, in response to hypoxia, the level of HIF-1α activation was markedly increased in AS compared to IS and controls (**Figure 1C**).

### Hypoxia Impaired Phagocytosis in Controls but Not in Sarcoidosis MD-Macrophages

**Figure 2:**
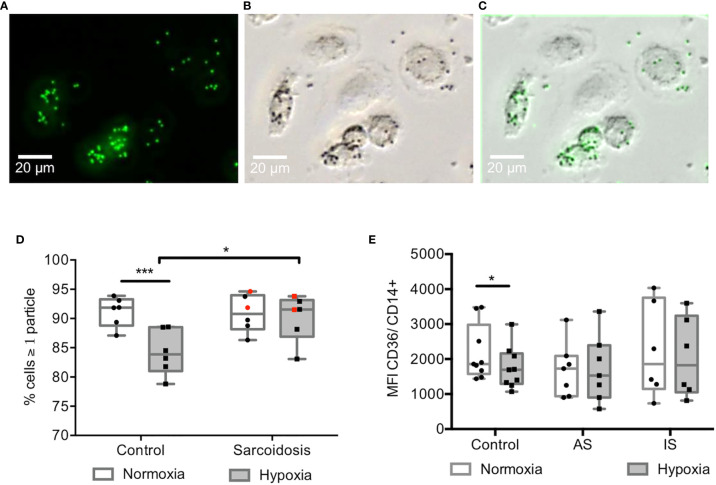
Hypoxia impaired phagocytosis in controls but not in sarcoidosis MD-macrophages. **(A–C)** Representative image of MD-macrophages exposed to normoxia from high active sarcoidosis after 30 min incubation with fluospheres (1µm) followed by four PBS washes (magnification x 200); **(A)** fluorescent 1µm fluospheres (green) **(B)**, phase-contrast images of MD-macrophages **(C)**, overlay images showing fluospheres within MD-macrophages. **(D)** Effect of hypoxia on phagocytosis in controls and sarcoidosis patients estimated by the ratio of cells with at least one particle and the total number of cells. Each point indicates a patient and/or control (n=6/group). In sarcoidosis group, black dots represent high active sarcoidosis (AS), and red dots low active or inactive sarcoidosis (IS); **(E)** Effect of hypoxia on the CD36 scavenger receptor expression in controls, AS, IS; analyzed by flow cytometry of CD14+ MD-macrophages. The results are expressed as the mean CD36 fluorescence intensity (MFI). Each point indicates a patient and/or control (n= 6-9/group). Results are expressed as box plot showing 25^th^ and 75^th^ percentile and median *p < 0.05; ***p < 0.001 in two-way ANOVA-repeated measures with Sidak *post-hoc* test.

It has been previously reported that phagocytosis, a main function of macrophages, can be modulated by HIF-1 (39) and involved in sarcoidosis pathogenicity (17, 40, 41). Therefore, phagocytosis was assessed in MD-macrophages exposed to normoxia or 24hrs-hypoxia and challenged with fluospheres (**Figures 2A–C**). In response to hypoxia, fluospheres phagocytosis was maintained in MD-macrophages from AS and IS, whereas it was decreased in controls (**Figure 2D**). Likewise, expression of the CD36 scavenger surface marker was maintained under hypoxic condition in MD-macrophages from sarcoidosis in contrast to controls (**Figure 2E**).

### Hypoxia Decreased CD80 and CD86 Co-Stimulation Molecules and Human Leucocyte Antigen-DR (HLA-DR)

**Figure 3:**
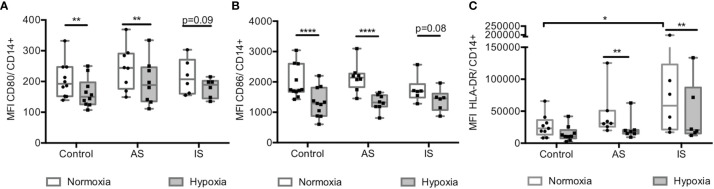
Hypoxia decreased CD80 and CD86 co-stimulation molecules and HLA-DR. **(A–C)** Effect of hypoxia on CD80, CD86 and HLA-DR surface expression on CD14+ MD-macrophages from controls, high active sarcoidosis (AS), and low active or inactive sarcoidosis (IS). Results are expressed as the mean fluorescence intensity (MFI) of CD80 **(A)**, CD86 **(B)** and HLA-DR **(C)** analyzed by flow cytometry. Results are expressed as box plot showing 25^th^ and 75^th^ percentile and median, each point indicates a patient and/or control (n = 6-11/group). *p < 0.05; **p < 0.01; ****p < 0.0001 in two-way ANOVA-repeated measures with Sidak *post-hoc* test.

Hypoxia effect on the expression of CD80 and CD86, co-stimulatory molecules interacting to activate T lymphocytes, and of the HLA-DR presenting antigen molecule were determined using flow cytometry. In normoxic condition, CD80 and CD86 were similarly expressed on MD-macrophages from all groups (**Figures 3A, B**). Hypoxia significantly decreased the expression of CD80 and CD86 on MD-macrophages from controls and AS (**Figures 3A, B**). A higher HLA-DR expression was observed in normoxic condition on MD-macrophages in IS compared with controls. Hypoxia significantly decreased HLA-DR expression on MD-macrophages only from sarcoidosis sub-groups (**Figure 3C**). By contrast, CD163 surface expression, did not vary on MD-macrophages from sarcoidosis and controls exposed to hypoxia (see **Figure E3**).

### Hypoxia Induced a Pro-Inflammatory Response Without Activation of NF-κB in High Active Sarcoidosis

**Figure 4:**
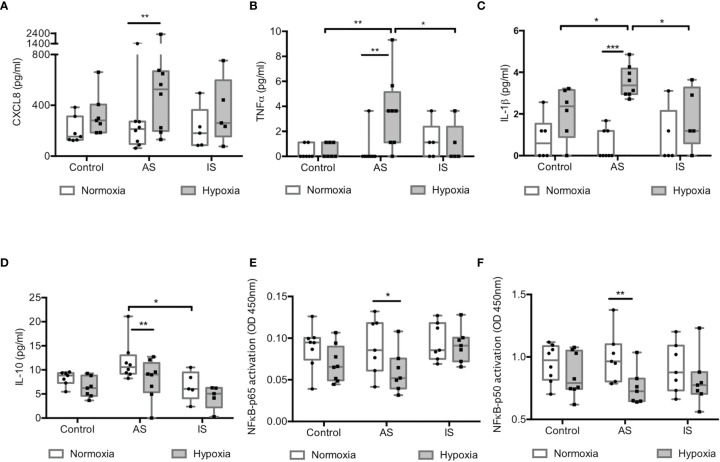
Hypoxia induced a pro-inflammatory response without activation of NF-κB in high active sarcoidosis. **(A–D)** Concentrations of CXCL8 **(A)**, TNFα **(B)**, IL-1ß **(C)** and IL-10 **(D)** assessed by Luminex^®^ in conditioned media of normoxic and hypoxic MD-macrophages from controls, high active sarcoidosis (AS), and low active or inactive sarcoidosis (IS); results are expressed in pg/ml (n= 5-8 controls or patients/group). **(E, F)** NF-κB-p65 and p50 activation (measured as OD450nm) assessed by TransAM ^®^. Each point indicates a patient and/or control (n= 7-8/group). Results are expressed as box plot showing 25^th^ and 75^th^ percentile and median. *p < 0.05; **p < 0.01; ***p < 0.001 in two-way ANOVA-repeated measures with Sidak *post-hoc* test.

Pro-inflammatory cytokine secretion in CM was compared in normoxia and hypoxia. Baseline concentrations of chemokine (C-X-C motif) ligand 8 (CXCL8), tumor necrosis factor-α (TNFα), interleukin-1ß (IL-1ß) (**Figures 4A–C**), IL-18 and IL-5 (see **Figure E4**) under normoxia were similar between groups. Hypoxia significantly increased pro-inflammatory cytokine secretion only in MD-macrophages from AS (**Figures 4A–C**). In normoxia, IL-10 secretion was increased in AS compared to IS, while hypoxia significantly reduced its secretion (**Figure 4D**). Increase in pro-inflammatory cytokine secretion induced by hypoxia in MD-macrophages from AS was associated with an increase in their mRNA levels (see **Figure E4**). The pro-inflammatory response to hypoxia was not associated with NF-κB (p65 and p50 subunits) activation which was decreased in AS (**Figures 4E, F**).

### Hypoxia Promoted a Profibrotic Response in High Active Sarcoidosis

**Figure 5:**
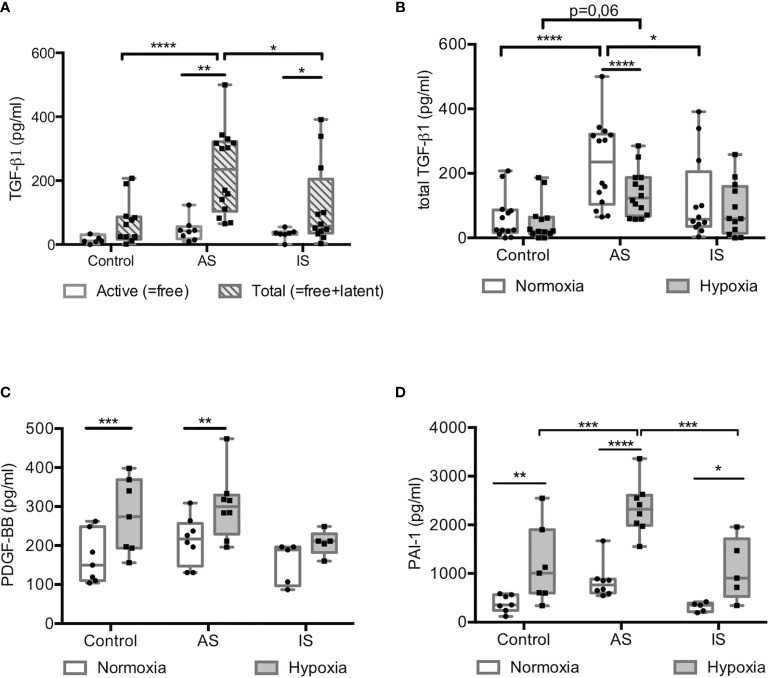
Hypoxia promoted a profibrotic response in high active sarcoidosis. **(A)** Active (free) and total (free+latent) TGß1 (in pg/ml) measured by ELISA in conditioned media from MD-macrophages after 24hrs of normoxia. (n= 7-14 independent experiments). **(B–D)** Concentrations of total TGFß1 **(B)**, PDGF-BB **(C)** and PAI-1 **(D)** assessed by Luminex^®^ in conditioned media of normoxic and hypoxic MD-macrophages from controls, high active sarcoidosis (AS), and low active or inactive sarcoidosis (IS). Each point indicates a patient and/or control (n= 5-8/group). Results are expressed in pg/ml with box plot showing 25^th^ and 75^th^ percentile and median. *p < 0.05; **p < 0.01; ***p < 0.001; ****p < 0.0001 in two-way ANOVA-repeated measures and two-way ANOVA with Sidak *post-hoc* test.

Secretion of profibrotic factors in CM of MD-macrophages, TGF-ß1, platelet-derived growth factor-BB (PDGF-BB), PAI-1 (**Figures 5A–D**) and vascular endothelial growth factor-A (VEGF-A) (see **Figure E5**) was studied. In normoxia, concentration of total TGF-ß1 was higher in CM from AS, especially the latent form of TGF-ß1 (**Figure 5A**). Hypoxia induced its decrease in AS, albeit remaining higher than in controls (**Figure 5B**). Likewise, hypoxia increased the secretion of PDGF-BB, PAI-1 (**Figures 5C, D**) and VEGF-A (see **Figure E5**) in all groups. These results were supported by an increase in mRNA transcript levels of profibrotic cytokines in MD-macrophages, especially *TGFB1* and *VEGF* in AS (see **Figure E5**).

### Secretion of PAI-1 by Hypoxic MD-Macrophages From High Active Sarcoidosis Inhibited Lung Fibroblast Migration

**Figure 6:**
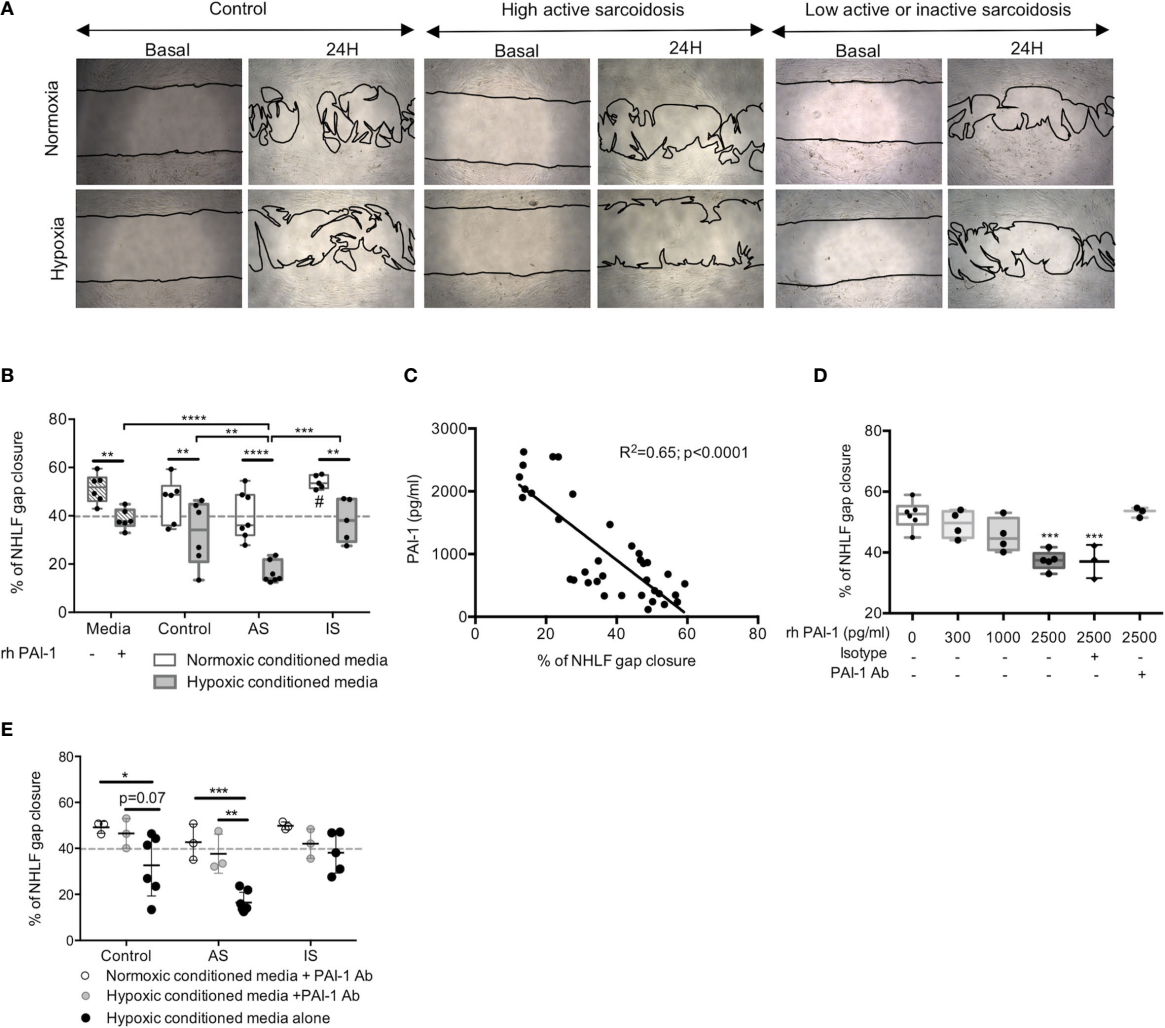
Secretion of PAI-1 by hypoxic MD-macrophages from high active sarcoidosis inhibited lung fibroblast migration. **(A)** Representative contrast-phase microscopy images of NHLF during gap closure assays at basal time and after 24hrs of incubation with conditioned media from controls or high active sarcoidosis (AS) or low active or inactive sarcoidosis (IS) patients MD-macrophages exposed to normoxia or hypoxia. **(B)** Quantitative analysis of NHLF gap closure assay comparing media alone or with 2500pg/ml recombinant human PAI-1 (rh-PAI-1) or normoxic or hypoxic conditioned media from MD-macrophages in controls, AS, and IS. Each point indicates a patient and/or control (n=5-7/group) **(C)** Correlation (Pearson Test) between PAI-1 level (pg/ml) in normoxic or hypoxic MD-macrophages conditioned media from sarcoidosis and controls and percentage of NHLF gap closure. Each point indicates a patient and/or control (n=3-6/group). **(D)** Dose effect of rh-PAI-1 on NHLF gap closure reversed by PAI-1 Ab (n=3 independent experiments); **(E)** Effect of PAI-1 antibody (PAI-1 Ab) added to the conditioned media on NHLF gap closure assay. Each point indicates a patient and/or control (n=3/group). Results are expressed as box plot showing 25^th^ and 75^th^ percentile and median **(B, D)** or mean with SD **(E)**. *p < 0.05; **p < 0.01; ***p < 0.001; ****p < 0.0001 in Anova two-way **(D, E)** and Anova two-way with repeated measures **(B)** with Sidak *post-hoc* test. ^#^p < 0.05 between normoxic conditioned media from AS and IS.

Among the profibrotic factors, PAI-1 secretion in hypoxia was two-fold higher in AS compared to IS and controls (**Figure 5D**). Accordingly, we hypothesized that PAI-1 may act on fibroblasts close to macrophages. The effect of CM from MD-macrophages exposed to either normoxia or hypoxia on gap closure in NHLF monolayers was studied. Exposure of NHLF to CM from hypoxia-exposed MD-macrophages from all groups significantly inhibited gap closure as compared with CM from normoxia-exposed MD-macrophages (**Figures 6A, B**). Hypoxic CM did not affect NHLF proliferation (see **Figure E6**) or fibroblast-myofibroblast differentiation as assessed by alpha-smooth actin and collagen type I quantification in Western blot (see **Figure E7**), indicating that inhibition of gap closure was related to a decrease in the ability of NHLF to migrate. The “immobilization” of NHLF was markedly observed with CM from hypoxic MD-macrophages from AS (**Figure 6B**).

Among the profibrotic factors secreted by MD-macrophages, PAI-1 levels negatively correlated with the percentage of NHLF gap closure (**Figure 6C**). Incubation of NHLF with increasing concentrations of rhPAI-1 reduced NHLF migration (**Figure 6D**). The inhibitory effect of rhPAI-1 on NHLF migration was prevented by addition of anti-PAI-1 antibody (**Figure 6D**). Inhibition of PAI-1 in the CM from hypoxic MD-macrophages with an anti-PAI-1 antibody restored NHLF migration, especially in AS (**Figure 6E**). These data indicate that PAI-1 secretion by hypoxic MD-macrophages from AS mostly contribute to inhibit human lung fibroblast migration.

### Detection of HIF-1α and PAI-1 in Granulomas From Archived Pulmonary Biopsies

**Figure 7:**
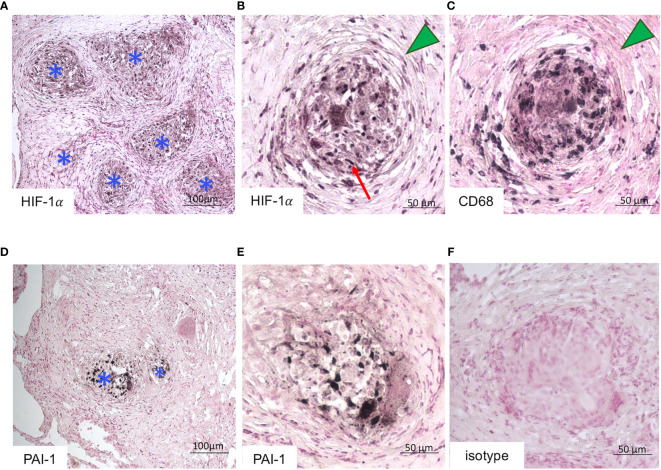
Detection of HIF-1α and PAI-1 in granulomas from pulmonary biopsies. **(A, B)** HIF-1α, **(C)** CD68 and **(D, E)** PAI-1 expression assayed by immunohistochemistry in archived lung biopsy from sarcoidosis patient. **(A–F)** are representative of the results obtained in lung biopsies from three sarcoidosis. **(A, D)** Granulomas are identified by a blue asterisks. **(B, C)** Serial sections of a granuloma wrapped with lamellar fibrosis (green triangle) showing HIF-1α expression in epithelioid cells characterized by their large cytoplasm, eccentric reniform nuclei (red arrow) **(B)** and CD68+ labelling **(C).** (**A, D** magnification x100 **B, C, E, F** magnification x200). Isotype control is shown in **(F)**.

We immunodetected HIF-1α (**Figures 7A, B**) and PAI-1 (**Figures 7D, E**) in granulomas from the three archived pulmonary biopsies. Macrophages-derived mature epithelioid cells were identified on their characteristic large cytoplasm, eccentric reniform nuclei (42) and CD68+ labelling (**Figures 7B, C**). Nuclear localization of HIF-1α and PAI-1 was detected in the mature epithelioid cells constituting granulomas (**Figures 7B, C, E**). An isotype control is shown in **Figure 7F**.

## Discussion

**Figure 8:**
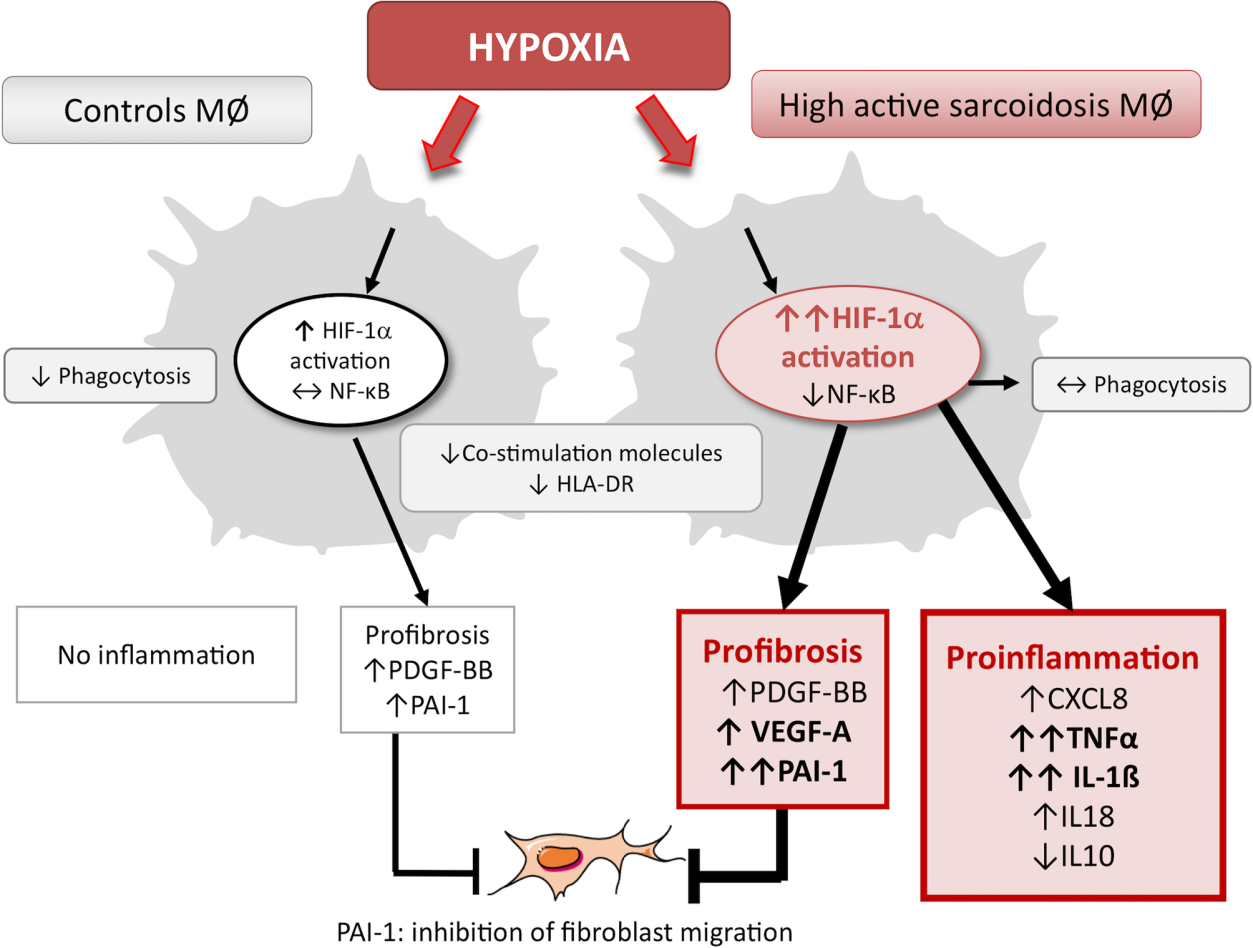
Summary of hypoxia biological impact on MD-macrophages from controls versus high active sarcoidosis. Hypoxia induced in MD-macrophages a significantly more pronounced effect in high active sarcoidosis compared with controls. It increased the HIF-1α trans-activity, promoted a proinflammatory response without activating NF-κB pathway and a profibrotic response (TGFß1, PDGF-BB) with PAI-1 secretion associated with human lung fibroblast migration inhibition. Hypoxia also decreased the expression of CD80/CD86 and HLA-DR on MD-macrophages in the two groups while it did not impair phagocytosis and the surface expression of CD36 on cells from high active sarcoidosis at variance with controls. MØ, MD-macrophages.

Factors modulating the onset and progression of sarcoidosis are still poorly known. Here we showed that hypoxia, a microenvironmental factor most likely present within granulomas and inflammatory tissues, activated HIF-1 and induced a mixed proinflammatory-profibrotic MD-macrophage phenotype particularly marked in AS, as illustrated in **Figure 8**. These *in vitro* results were supported by immunohistochemistry data showing the expression of HIF-1α and its target PAI-1 in epithelioid cells constituting pulmonary sarcoidosis granulomas.

One strength of our study was to prospectively include patients with either high active or low active or inactive sarcoidosis, not taking any therapy that could modify macrophage biology. The aCTAS score we used to evaluate the disease activity has already been validated by two independent studies (32, 43). This score was highly correlated with the range of FVC improvement under further sarcoidosis treatment, a good marker of the lesions reversibility. The choice to specifically study MD-macrophages in these patients was supported by recent works showing that *in vitro* models of granuloma using PBMCs can mimic sarcoidosis events such as phagosome-regulated mTOR or IL-13 signaling (17, 44). In addition, previous studies reported that immune cells constituting recurrent sarcoidosis granulomas in lung transplant allografts originated from the recipient monocyte-macrophage lineage (19, 20). Finally, the inflammatory phenotype of MD-macrophages in pulmonary sarcoidosis and their propensity to produce TNFα has been recently highlighted (11), supporting the role of these cells in sarcoidosis pathogenesis. However, the fact that experiments were almost exclusively performed in MD-macrophages constitutes a limitation of the present study. It would certainly be interesting to study the effects of hypoxia on other cell types involved in sarcoidosis such as T lymphocytes. A comparison of our data in MD-macrophages with data obtained in lung resident alveolar macrophages could also be informative but bronchoalveolar lavages are rarely done in the follow-up of sarcoidosis patients. Another limitation of our study is the fact that it was not possible to conduct all experiments for each patient for technical reasons, i.e. relatively to the low number of retrieved monocytes per blood sample.

To the best of our knowledge, the consequences of a hypoxic microenvironment on macrophages were never investigated in sarcoidosis, although granuloma hypoxia was inferred from morphometric analysis (24). By contrast, in tuberculosis pulmonary granulomas, hypoxia was directly demonstrated to play a major role in the disease course (25, 45). Recently, 18F-fluoromisonidazole uptake, a hypoxia-sensitive PET tracer, was observed in sarcoidosis lesions and associated with ^18F^FDG-PET uptake (35), suggesting that hypoxic lesions are metabolically active and associated with an active form of sarcoidosis. In addition, patients with high active sarcoidosis had more impaired lung function with lower DLCO, potentially resulting in local alveolar hypoxia.

Previous studies investigating HIF in sarcoidosis led to conflicting results (28, 29). Tzouvelekis et al. (29) reported increased expression of VEGF, a target of HIF, within sarcoidosis granulomas but failed to detect HIF-1α whereas Talreja et al. did (28). Such a difference in HIF-1α expression detection could be explained by different stages in the disease between the studies, the different antibodies used for HIF-1α detection and immunosuppressive treatments given to patients in the Tzouvelekis et al. study (28, 29). However, in line with Talreja et al. (28), we clearly immunodetected HIF-1α in pulmonary sarcoidosis granulomas. Such a finding prompted us to evaluate *in vitro* the effects of hypoxia on MD-macrophages from controls and untreated sarcoidosis patients.

In the present study HIF-1α nuclear expression was detected under normoxia in MD-macrophages from patients and controls. Talreja et al. also reported HIF-1α expression in freshly-isolated blood monocytes (28). The nuclear expression of HIF-1α in normoxic MD-macrophages is consistent with the fact that non-hypoxic stimuli such as TNF-α and IL-1β proinflammatory cytokines, NF-κB, reactive oxygen species, MAPK (46, 47) or mTOR pathway activation (48) are able to stabilize HIF-1α in immune cells. However, HIF-1α transcriptional activity was significantly more strongly induced by hypoxia in MD-macrophages from AS. Consistently, several HIF-target genes were induced by hypoxia in MD-macrophages from AS, such as *VEGF* and *TGFB1* transcripts or PAI-1 protein. The TNFα and IL-1ß cytokines [involved in sarcoidosis pathogenesis (4, 49)] and CXCL8, induced by hypoxia only in AS, can also be directly upregulated by HIF in macrophages (50, 51). NF-κB activation, known to be also induced by hypoxia and leading to pro-inflammatory cytokine production (52) was decreased in AS, suggesting that it was not involved in this context. Moreover, HIF-1 can exert a negative feedback on NF-κB (53). The stronger response to hypoxia observed in MD-macrophages from AS is somewhat intriguing. It might be explained by still unexplored genetic polymorphisms, by epigenetic mechanisms and/or non-coding RNAs known to modulate HIF expression (54).

We investigated whether hypoxia could modulate phagocytosis and antigen processing/presentation, a key initial step of inflammatory lesions in sarcoidosis. We observed that phagocytosis was similar at baseline in control and sarcoidosis MD-macrophages. Interestingly, hypoxia markedly reduced phagocytosis in controls but not in sarcoidosis, suggesting that macrophage phagocytosis could be maintained under hypoxia in this disease. Previous papers reported that phagocytic activity of macrophages was increased in sarcoidosis and in an *in vitro* model of granuloma (17, 40, 41). An enhanced expression of phagocytosis-related genes, but a downregulation of genes involved in proteasome degradation were also observed, suggesting that the accumulation of intracellular phagocytic degradation products could participate in the chronicity of inflammation in sarcoidosis (40). We next evaluated the effect of hypoxia on the expression of CD80 and CD86 co-stimulatory molecules and on HLA-DR as these markers are associated with antigen presentation and expressed by epithelioid cells in sarcoidosis granuloma (55, 56). Hypoxia markedly decreased the expression of CD80 and CD86 on MD-macrophages in controls and AS while not significantly in IS. Hypoxia also downregulated the expression of HLA-DR in AS and IS MD-macrophages. These findings are in line with previous studies showing that hypoxia decreased the expression of CD80 in murine macrophages and CD80, CD86 and MHC class II in human dendritic cells, reducing their ability to initiate adaptive-immunity responses (57, 58). However, the role of antigenic presentation in sarcoidosis is still debated. Crouser et al. reported an enhanced presentation capacity in an *in vitro* model of sarcoidosis (17). Conversely, Grunewald et al. proposed that granuloma progression may occur when antigen recognition is not efficient enough, due to inadequate peptide presentation by modified HLA molecules or T cells incapacity to generate T cell clones, allowing granuloma persistence because of inefficient adaptive immune response (4). Here, we can speculate that hypoxia at the various steps towards granuloma formation may potentially exert a deleterious effect on antigen presentation processes, and therefore contribute to their persistence.

Mechanisms leading to sarcoidosis-associated pulmonary fibrosis are poorly understood. Granulomas are almost always surrounded by a concentric rim of collagen bundles (3). Macrophages are known to be involved in repair and fibrotic processes through different states of polarization (59). In sarcoidosis, macrophages may acquire a profibrotic M2-phenotype as shown in granulomas from patients’ biopsies or *in vitro* model (44, 60). In our study, hypoxia decreased the expression of M1 markers (CD80, CD86, HLA-DR), while M2 markers expression was maintained (CD163, CD36). However, hypoxia was also associated with a M1 pro-inflammatory cytokine response. In the literature, the effect of hypoxia on macrophage polarization remains unclear (61, 62). More than the over-simplified M1/M2 dichotomy, macrophages display remarkable plasticity and can change their phenotype in response to environmental factors.

Previous studies showed that under hypoxic conditions, macrophages can induce fibrosis in a HIF-dependent manner through the secretion of VEGF, PDGF-BB, or PAI-1 (63, 64). Here, we observed that MD-macrophages from AS secreted higher levels of PAI-1 in response to hypoxia as compared with controls or IS. The PAI-1 factor plays a key role in the development of pulmonary fibrosis by fibrotic matrix deposition (65). An increase in PAI-1 levels as well as a decrease in fibrinolytic activity has been reported in the bronchoalveolar lavage of patients with sarcoidosis (66). We clearly detected PAI-1 in granuloma epithelioid cells. Finally, we demonstrated that CM from hypoxic MD-macrophages inhibited pulmonary fibroblast migration, a phenomenon highly dependent on PAI-1. Consistently, PAI-1 binding to vitronectin has been previously shown to inhibit cell migration, leading to inefficient alveolar repair following injury and favoring the development of pulmonary fibrosis (67). Therefore, we hypothesize that PAI-1 secretion by granuloma cells could immobilize and sequester lung fibroblasts around granuloma, thus favoring the characteristic peripheral granuloma fibrosis.

In conclusion, this study shows that hypoxia exerts a significant and specific impact on MD-macrophages from sarcoidosis patients, with the strongest effect observed in patients with a high active disease. It may favor the development and persistence of granulomas in active sarcoidosis and fibrosis surrounding granulomas by promoting a mixed inflammatory/fibrosing response of macrophages, by reducing their antigen presentation capacities, leading to a deficient T cell response. Thus, the HIF pathway and PAI-1 could be involved in the pathogenesis of high active sarcoidosis, potentially representing new therapeutic targets. As a future direction, we plan to investigate interactions between HIF and other signaling pathways identified in the pathogenesis of sarcoidosis as mTOR, NLRP3 inflammasome, JAK/STAT, or heat shock proteins (49, 68, 69), already known to interfere with the HIF/hypoxia pathway (22, 70–72).

## Data Availability Statement

The original contributions presented in the study are included in the article/**Supplementary Material**. Further inquiries can be directed to the corresponding author.

## Ethics Statement

The studies involving human participants were reviewed and approved by Comité de protection des personnes (CPP) Ile-de-France X 2016-10-02. The patients/participants provided their written informed consent to participate in this study.

## Author Contributions 

FJ and VB designed research studies, contributed to the experiments, analyzed the data, and wrote the manuscript. HN and MK provided human samples and reviewed the manuscript. MP contributed to the experiments and reviewed the manuscript. CP, J-FB, DV, and HN wrote the manuscript. All authors contributed to the article and approved the submitted version.

## Funding

This work was supported by: Fonds de Recherche en Santé Respiratoire et Fondation du Souffle (2017), Financement de la Chancellerie des Universités de Paris-Legs Poix (2017), Bonus Qualité Recherche Université Sorbonne Paris- Nord (2016-2017).

## Conflict of Interest

The authors declare that the research was conducted in the absence of any commercial or financial relationships that could be construed as a potential conflict of interest.

## Publisher’s Note

All claims expressed in this article are solely those of the authors and do not necessarily represent those of their affiliated organizations, or those of the publisher, the editors and the reviewers. Any product that may be evaluated in this article, or claim that may be made by its manufacturer, is not guaranteed or endorsed by the publisher.
