# Supplementary material for: Hypoxia Promotes a Mixed Inflammatory-Fibrotic Macrophages Phenotype in Active Sarcoidosis
Source: Front Immunol. 2021 Aug 11;12:719009. doi: 10.3389/fimmu.2021.719009 (PMC8385772; doi:10.3389/fimmu.2021.719009)
Supplement: Supplementary file 1 [file DataSheet_1.docx]

**Online Data Supplement**

**Hypoxia promotes a mixed inflammatory-fibrotic macrophages phenotype in active sarcoidosis**

Florence Jeny, Jean-François Bernaudin, Dominique Valeyre, Marianne Kambouchner, Marina Pretolani, Hilario Nunes, Carole Planès, Valérie Besnard

**Number of supplemental figures**: 7

**Number of supplemental tables:** 2

**Supplemental methods**

1. **Patients and controls**

In this study, any patient with an abbreviated Computed Tomography Activity Score aCTAS (1) ≥2 was considered to have an active disease (fig E1). In case of lung fibrotic sarcoidosis, a significant increased uptake (higher than liver uptake) on ^18F^FDG PET-CT scan on parenchyma was required to classify patient with active pulmonary disease.

The medical records of all patients were reviewed (FJ) and data regarding demographics, radiographic stages, pulmonary function tests (spirometry and DLCO), chest CT ± ^18F^FDG PET-CT scan and extra-pulmonary organ involvements (according to clinical and CT data) were recorded at the time of the blood draws.

# Peripheral blood mononuclear cells isolation, culture and macrophage differentiation

Twenty mL of total blood sampled in EDTA tubes for routine hematology analysis were collected. Peripheral blood mononuclear cells (PBMCs) were isolated using Ficoll-Paque^TM^ plus (GE healthcare #17-1440-03) (density 1.077 g/ml) density gradient separation. Monocytes were purified from human PBMCs using the Pan Monocyte Isolation Kit (Miltenyi Biotec, France #130-096-537) according to the manufacturer’s instructions, and then seeded in non-coated 12-well plates or 6-well plates at 1x10^6^ and 0.5x10^6^ cells/well respectively, in RPMI medium 1640 GlutaMAX^TM^ containing 2 mM of glutamine, 25 mM of HEPES (Gibco #72400021), fetal bovine serum 10% (Gibco #10270), penicillin/streptomycin (Gibco #15140-122). Monocytes were differentiated into macrophages in presence of 5ng/ml M-CSF (BioTechne, France #216-MC) for 10 days in 5% CO_2_ -95% air atmosphere (*i.e*. 21% O_2_). Medium was changed every 3 days. Purity of enriched monocytes was evaluated by flow cytometry using APC-Cy^TM^7 mouse anti-Human CD14 (BD Biosciences #557831); the purity of monocytes > 90%.

1. **Normoxic and hypoxic culture conditions**

Cell cultures under hypoxic conditions were undertaken in a hermetic humidified chamber (Saint Bernard Technologies, AYDAT) equilibrated with a gas mixture containing 1.5% O_2_ - 5% CO_2_ - 93.5 % N_2_ (oxygen tension in culture media: 45 mmHg), and kept at 37°C for 24 hours). Normoxic control cells were maintained in a 21% O_2_ - 5% CO_2_ - 74% N_2_ humidified incubator for the same period of time (oxygen tension in culture media: 140 mmHg).

1. **Flow cytometry and cell surface staining**

Monocyte-derived macrophages were stained for cell surface markers using fluorescent labelled antibodies for CD14, CD80, CD86, HLA-DR, CD36, CD163 and isotypes-matched IgG (*see* Table E2). Cells were detached with PBS-EDTA 1mM, washed with PBS-FBS 2% and incubated with antibodies or isotypes-matched IgG for 30min at 4°C in PBS solution containing FBS 2% and bovine serum albumin 5% (sigma #A7030). Compensation between the different fluorescent antibodies was done before experiments using compensation particles set (BD Biosciences #552843). At least 20 000 cells per sample were analyzed on a flow cytometer on Canto II, BD Biosciences and data analysis was performed using BD FACS Diva^TM^ software and FlowJo^TM^ v10.6. Samples were gated on cells using FSC/SSC and doublet discrimination to identify singlets using SS-W vs SS-A, monocyte-derived macrophages were identified on the basis of CD14+ expression (see Fig E2).

1. **Immunofluorescent staining**

Monocyte-derived macrophages were plated on Labtek 8 well-chambers slide (Ibidi, France) at a density of 0.2 10^6^ cells/well and placed either under control atmosphere or under hypoxia for 24hrs. Cells were washed three times with cold PBS and fixed in 4% paraformaldehyde containing phosphate-buffered saline. After fixation, slides were placed in blocking buffer (PBS containing Triton X-100 0.1% BSA 1%). After washing, slides were incubated overnight at 4°C in a humidified chamber with an anti-HIF1α rabbit antibody (HIF1alpha (Novus) # NB100479). Control staining included cells incubated with 1% BSA and rabbit IgG 0.5 μg/ml (Vector Laboratories, USA). After being rinsed, slides were incubated with Alexa-fluor 568-conjugated goat anti-rabbit antibody (# A11036 Invitrogen) for 30 minutes, rinsed, counterstained with DAPI and mounted with Vectashield (Vector Laboratories, USA). To detect hypoxia in cells, 400µM pimonidazole (Hypoxyprobe ^TM^) was added in the medium for 2hrs, then cells were immunostained as described above using an anti-pimonidazole mouse IgG1 monoclonal antibody (MAb1) (1/50 Hypoxyprobe ^TM^ kit) overnight at 4°C with 5% BSA. The secondary antibody used was Alexa-fluor 488- conjugated goat anti-mouse antibody (#A11029 Invitrogen).

1. **Fibroblast gap-closure assays, proliferation assays**

Normal Human Lung Fibroblasts, NHLF (Lonza) were grown in Dulbecco’s Modified Eagle Medium (DMEM) supplemented with 2mM L-glutamine, 100 U/ml penicillin, 100µg/ml streptomycin, 10µg/ml gentamycin, 10% FBS. NHLFs were used at the 6-9 passages and seeded in non-coated 96-well plates at 3x10^4^ cells/well.

Cell migration was monitored by recording phase-contrast images (3 images, objective x50) along the gap at 0hr and 24hrs. Image J software was used to measure the gap area, and percentage of gap-closure was estimated by the difference between initial area and final area at 24Hrs relative to initial area.

For NHLF proliferation, cells were cultured in conditioned media of MD-macrophages for 24hrs and fixed with 4% paraformaldehyde. Cells were immunostained as described above using an anti-Ki67 antibody (#14-5698-82 invitrogen) overnight at 4°C. The secondary antibody used was Alexa-fluor 555- conjugated goat anti-rat antibody. Slides were counterstained with DAPI and mounted with Vectashield (Vector Laboratories, USA). Proliferation index was estimated by the number of Ki67+ cells/relative to the total number of cells using Image J software on 2 fields along the gap and outside the gap (objective x50).

1. **Western Blot**

Fibroblast-myofibroblast differentiation was assessed by alpha-smooth actin (αSMA) and collagen type I (COL1) quantification in Western blot. NHLF were seeded in 24-well plates at 12x10^4^ cells/well, and cultured either with conditioned media of MD-macrophages or RPMI FBS10% ± TGFß1 at 1ng/ml for 24hrs.

Protein expression was quantified by Western blot analysis in whole-cell homogenate. Proteins were extracted by addition of lysis buffer (250 mM NaCl, 50 mM HEPES pH 7.0, 5 mM EDTA, 1 mM dithiothreitol [DTT], 0.1% nonidet NP40, 10 μg/ml aprotinin, 10 μg/ml leupeptin, 50 μg/ml phenylmethylsulfonyl fluoride, 2 mM sodium pyrophosphate, and 1 mM sodium orthovanadate), diluted in Laemmli buffer, and subjected to SDS-PAGE under reducing condition. Immunostaining was performed with specific antibodies (goat anti-COL1 (Southern Biotech, 1310-01) (1/1000), mouse anti αSMA (Sigma Aldrich A2547) (1/4000), rabbit anti- beta-Tubulin (Santa-Cruz sc-9104) 1/500) according to manufacturer’s protocols. Protein ratios were normalized to the corresponding expression level of anti-β tubulin, used as a loading control. The signals were quantified by densitometric analysis using NIH image software, normalized using quantification of the beta-tubulin signal in each lane and expressed in arbitrary units.

**References:**

1. Benamore R, Kendrick YR, Repapi E, Helm E, Cole SL, Taylor S, et al. CTAS: a CT score to quantify disease activity in pulmonary sarcoidosis. Thorax. 2016;71(12):1161‑3.

**Figure Legends**

**Figure E1: Examples of abbreviated Computed Tomography Activity Score (aCTAS)**

A) patient with an aCTAS=0; B) patient with an aCTAS=1, with presence of micronodules (red arrows) ; C) patient with aCTAS=2, with presence of micronodules (red arrow) and interlobular septal thickening (yellow arrows) (D-E) patients with an aCTAS=3, with D) micronodules (red arrows), interlobular septal thickening (yellow arrow), ground glass opacification (*) or E) with consolidation (#), micronodules and nodules (red arrow) and interlobular septal thickening (yellow arrows).

**Figure E2: Cytometry data**

A) Pseudocolor plots and histograms from monocytes-derived macrophages of one representative high active sarcoidosis in normoxia. This figure illustrates the gating strategy used to identify CD14+ cells in order to analyze CD36, CD163, CD80, CD86, HLA-DR markers in cytometry. B) Effect of hypoxia 1,5% O_2_ (in blue) versus normoxia 21% O_2_ (in red) on the expression of surface marker on CD14+ MD-macrophages in one representative high active sarcoidosis. Histograms depict the results obtained**.**

**Figure E3: Hypoxia did not change the expression of CD163**

Effect of hypoxia on the CD163 expression in controls, high active sarcoidosis (AS), and low active or inactive sarcoidosis (IS). Results are expressed as the mean fluorescence intensity (MFI) of CD163 surface marker on CD14+ MD-macrophages using flow cytometry. Results are expressed as box plot showing 25^th^ and 75^th^ percentile and median, each point indicates a patient and/or control (n= 5-8/group) and analyzed with two-way ANOVA-repeated measures with Sidak post-hoc test.

**Figure E4:** **Hypoxia induced a proinflammatory response**

**(A-B)** Concentrations of IL-18 (A), IL-5 (B) as assessed by Luminex® in conditioned media of normoxic and hypoxic MD-macrophages from controls, high active sarcoidosis (AS), and low active or inactive sarcoidosis (IS); results are expressed in pg/ml. Each point indicates a patient and/or control (n= 5-8/group). **(C-G)** Effect of hypoxia on the transcription level assessed by RTqPCR of (C) *IL1B,* (D) *CXCL8,* (E) *IL18,* (F) *IL10,* (G) *TNFA* in MD-macrophages (n= 4-10 independent experiments)*.* Results are expressed with box plot showing 25^th^ and 75^th^ percentile and median. * p<0.05; **p<0.01; ***p<0.001 in two-way ANOVA-repeated measures with Sidak post-hoc test. *UBC=* ubiquitin C

**Figure E5:** **Hypoxia promoted a profibrotic response**

**(A)** Concentrations of VEGF-A as assessed by Luminex® in conditioned media of normoxic and hypoxic MD-macrophages from controls, high active sarcoidosis (AS), and low active or inactive sarcoidosis (IS); results are expressed in pg/ml. Each point indicates a patient and/or control (n= 5-8/group). (B-D) Effect of hypoxia on the transcription level assessed by RTqPCR of (B) *VEGF,* (C) *PDGFB,* (D) *TGFB1* in MD-macrophages. (n= 4-9/group)*.* Results are expressed with box plot showing 25^th^ and 75^th^ percentile and median. * p<0.05; ***p<0.001; in two-way ANOVA-repeated measures with Sidak post-hoc test. *UBC=* ubiquitin C

**Figure E6: No effect of conditioned media from MD-macrophages on NHLFs proliferation**

Effect of conditioned media from MD-macrophages on NHLF proliferation studied by anti Ki67 immunolabelling. (A-C) representative image (magnification x 200) of Ki67 staining in red (A), DAPI (B) and overlay image (C) showing nuclear localization of Ki67 in NHLF. (D) Proliferation index was estimated by the number of Ki67+ cells/relative to the total number of cells. Each point indicates a patient and/or control (n=5-6/group). “Media” consists of RPMI medium and 10%FBS. High active sarcoidosis (AS), and low active or inactive sarcoidosis (IS). Results are expressed with box plot showing 25^th^ and 75^th^ percentile and median. No significant difference in two-way ANOVA-repeated measures with Sidak post-hoc test.

**Figure E7: No effect of conditioned media from MD-macrophages on fibroblast-myofibroblast differentiation**

Effect of conditioned media from MD-macrophages on fibroblast to myofibroblast differentiation studied by expression of collagen type I (COL1) and alpha smooth muscle actin (alphaSMA) in Western blot. “Media” consists of RPMI medium and 10%FBS with or without recombinant human TGFß1 (rh TGFß1) at 1ng/ml (A-C). High active sarcoidosis (AS), and low active or inactive sarcoidosis (IS). Results are expressed with box plot showing 25^th^ and 75^th^ percentile and median. * p<0.05; in two-way ANOVA-repeated measures with Sidak post-hoc test (A, B). Each point indicates a patient and/or control (n=5-7/group). Representative immunoblot for COL1 and alphaSMA from NHLF cultured with (Nx) normoxic conditioned media and (Hx) hypoxic conditioned media from MD-macrophage from control, AS and IS(C).

**Table E1**: QPCR primers list.

| **Gene** | **Forward primer (5’- 3’)** | **Reverse primer (5’- 3)** |
| --- | --- | --- |
| *UBC* | CACTTGGTCCTGCGCTTGA | TTTTTTGGGAATGCAACAACTT |
| *CXCL8* | AGGACAAGAGCCAGGAAGAA | ACTGCACCTTCACACAGAGC |
| *TNF-A* | CCCATGTTGTAGCAAACCCT | TGAGGTACAGGCCCTCTGAT |
| *IL-1B* | AATCTGTACCTGTCCTGCGTGTT | TGGGTAATTTTTGGGATCTACACTCT |
| *IL-18* | GGGAAGAGGAAAGGAACCTC | CCATCTTTATTCCTGCGACA |
| *IL-10* | TGAGAACCAAGACCCAGACA | AAAGGCATTCTTCACCTGCT |
| *CXCL8* | AGGACAAGAGCCAGGAAGAA | ACTGCACCTTCACACAGAGC |
| *PDGFB* | TCGATCCGCTCCTTTGATGA | GGAACCCAGGCTCCTTCTTC |
| *VEGF* | CTCCACCATGCCAAGTGGTC | TCCACCAGGGTCTCGATTGG |
| *TGFB1* | TTTGATGTCACCGGAGTTGT | TAGTGAACCCGTTGATGTCC |

**Table E2:** list of cell surface markers using fluorescent labelled antibodies

| Company | Antibody/Isotype | Fluorochrome | clone | Reference |
| --- | --- | --- | --- | --- |
| BD Biosciences | Mouse, Anti-Human CD14 | APC-Cy^7^ | MϕP9 | 557831 |
| BD Biosciences | Mouse, IgG2b, κ | APC-Cy^7^ | 27-35 | 558061 |
| BD Biosciences | Mouse, Anti-Human CD86 | PE-Cy^7^ | 2331 | 561128 |
| BD Biosciences | Mouse IgG1, κ | PE-Cy^7^ | MOPC-21 | 557656 |
| BD Biosciences | Mouse anti-Human HLA-DR | APC | TU36 | 559868 |
| BD Biosciences | Mouse IgG2b, κ | APC | 27-35 | 555745 |
| BD Biosciences | Mouse anti-Human CD36 | APC | CB38 | 550956 |
| BD Biosciences | Mouse IgM, κ isotype control | APC | G155-228 | 555585 |
| BD Biosciences | Mouse anti-Human CD80 | PE | L307.4 | 557227 |
| BD Biosciences | Mouse IgG1, κ | PE | MOPC-21 | 554680 |
| BD Biosciences | Mouse Anti-Human CD163 | FITC | GHI/61 | 563697 |
| BD Biosciences | Mouse IgG1, κ | FITC | MOPC-21 | 555909 |
