# Supplementary figures and images for: Hypoxia Promotes a Mixed Inflammatory-Fibrotic Macrophages Phenotype in Active Sarcoidosis
Source: Front Immunol. 2021 Aug 11;12:719009. doi: 10.3389/fimmu.2021.719009 (PMC8385772; doi:10.3389/fimmu.2021.719009)

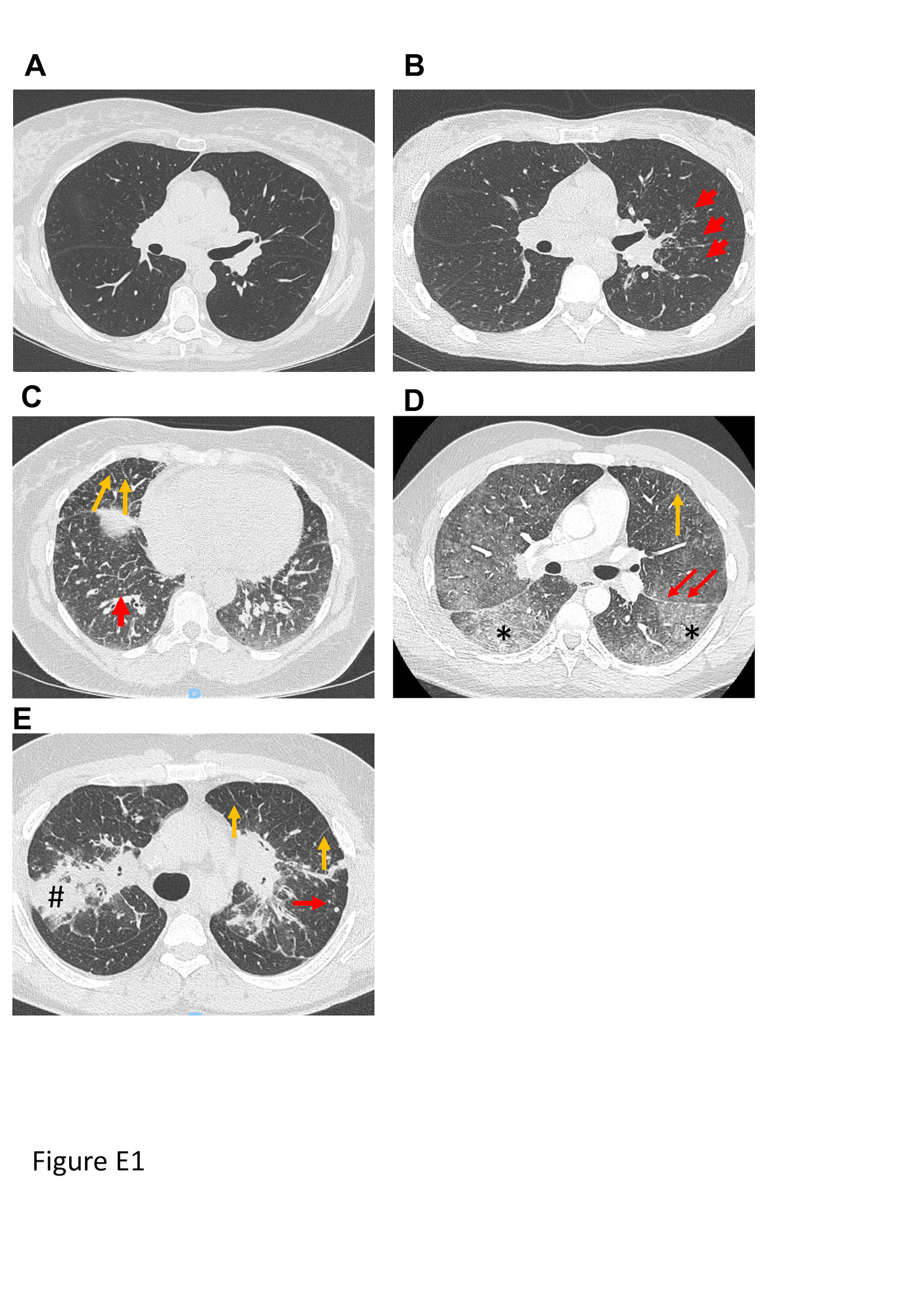

Supplement: Supplementary file 2 [file Image_1.tiff]

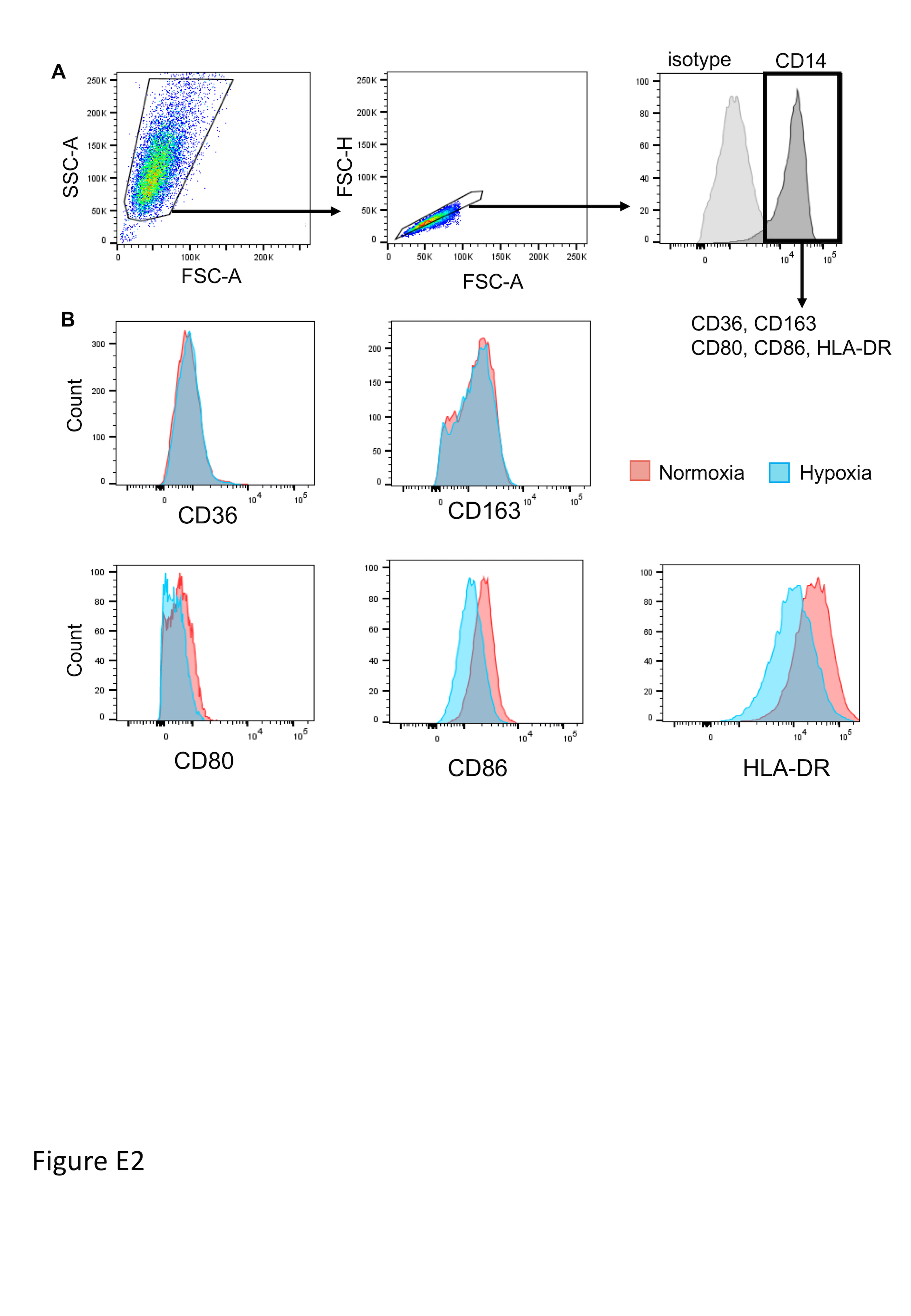

Supplement: Supplementary file 3 [file Image_2.tiff]

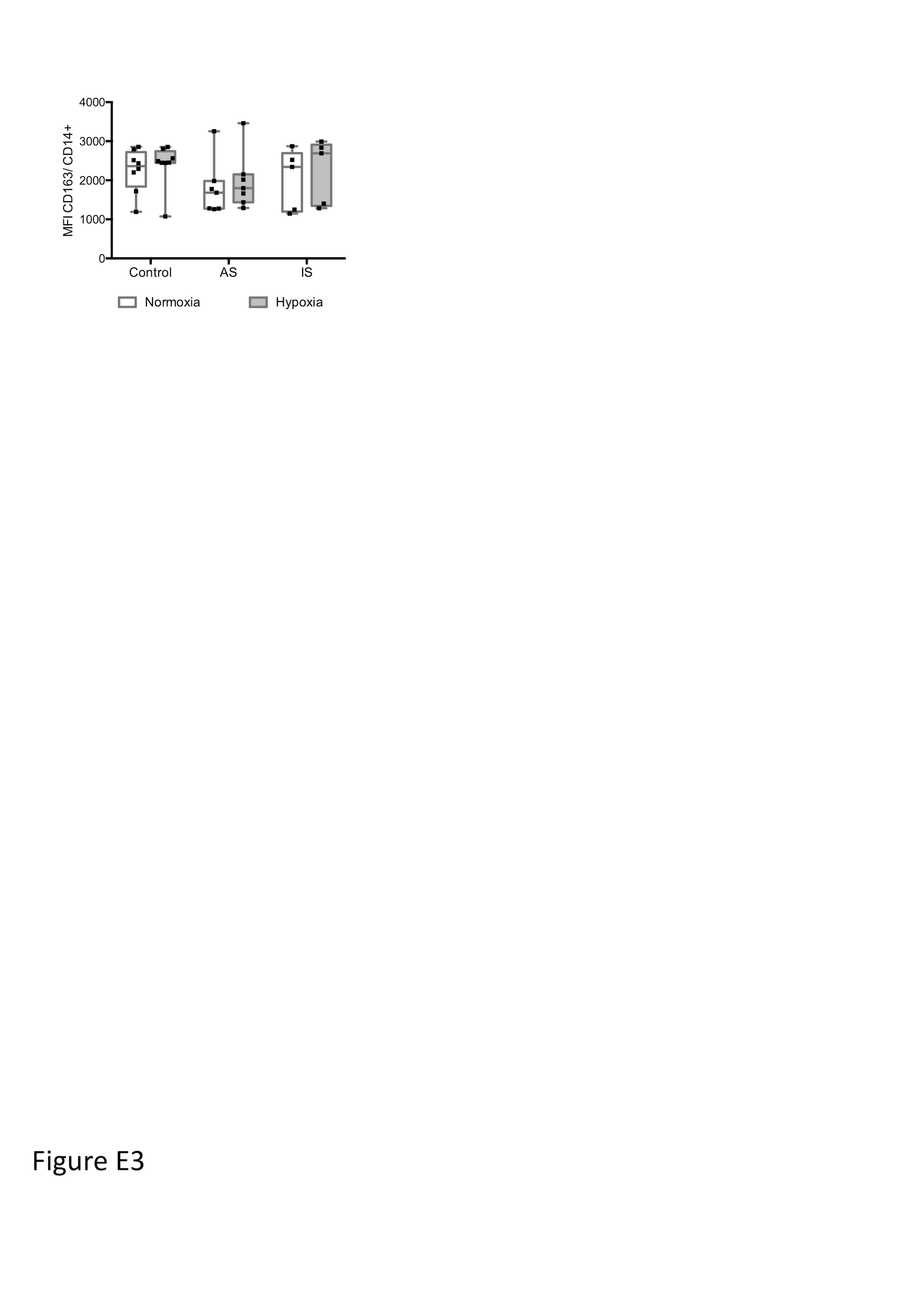

Supplement: Supplementary file 4 [file Image_3.tiff]

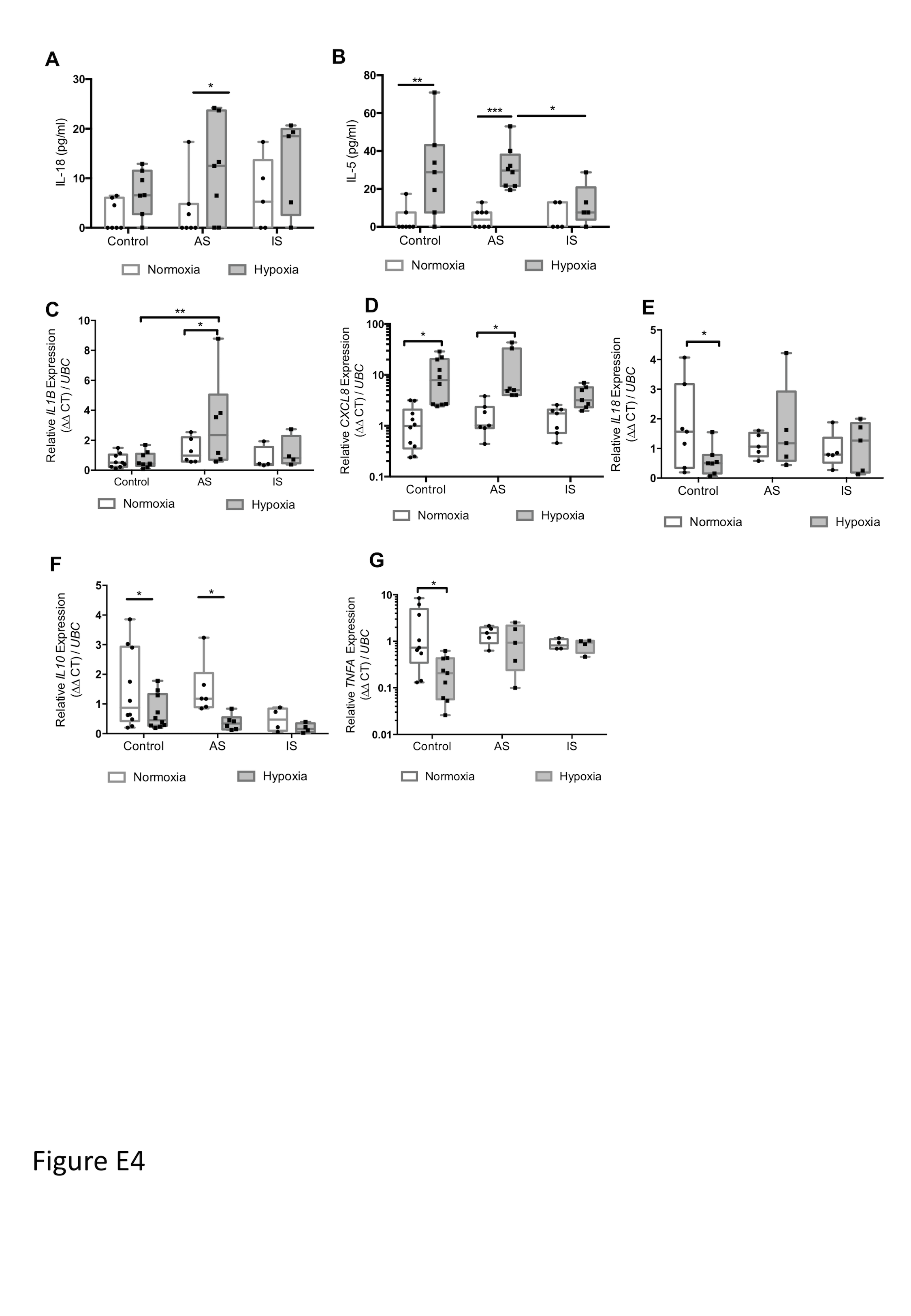

Supplement: Supplementary file 5 [file Image_4.tiff]

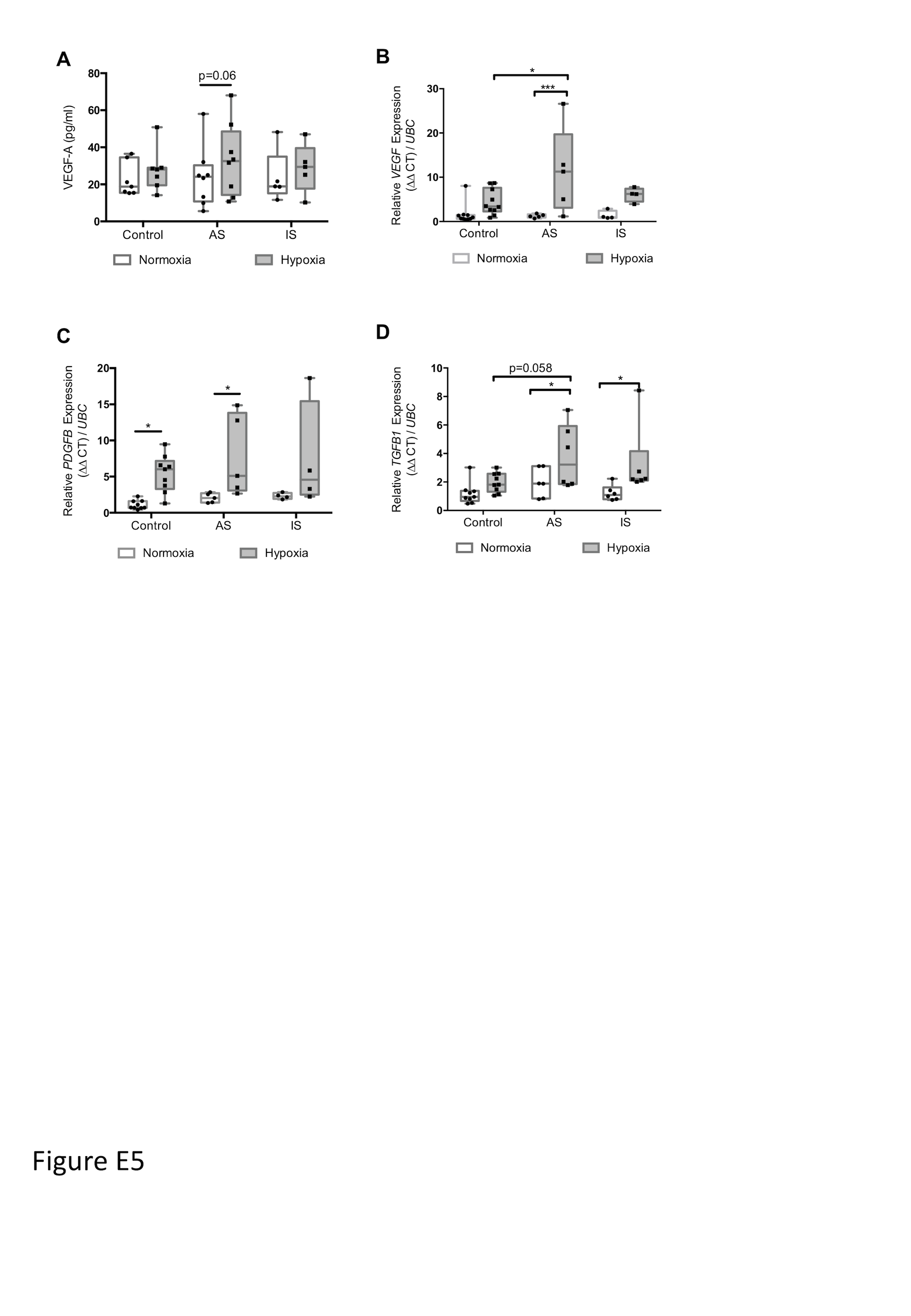

Supplement: Supplementary file 6 [file Image_5.tiff]

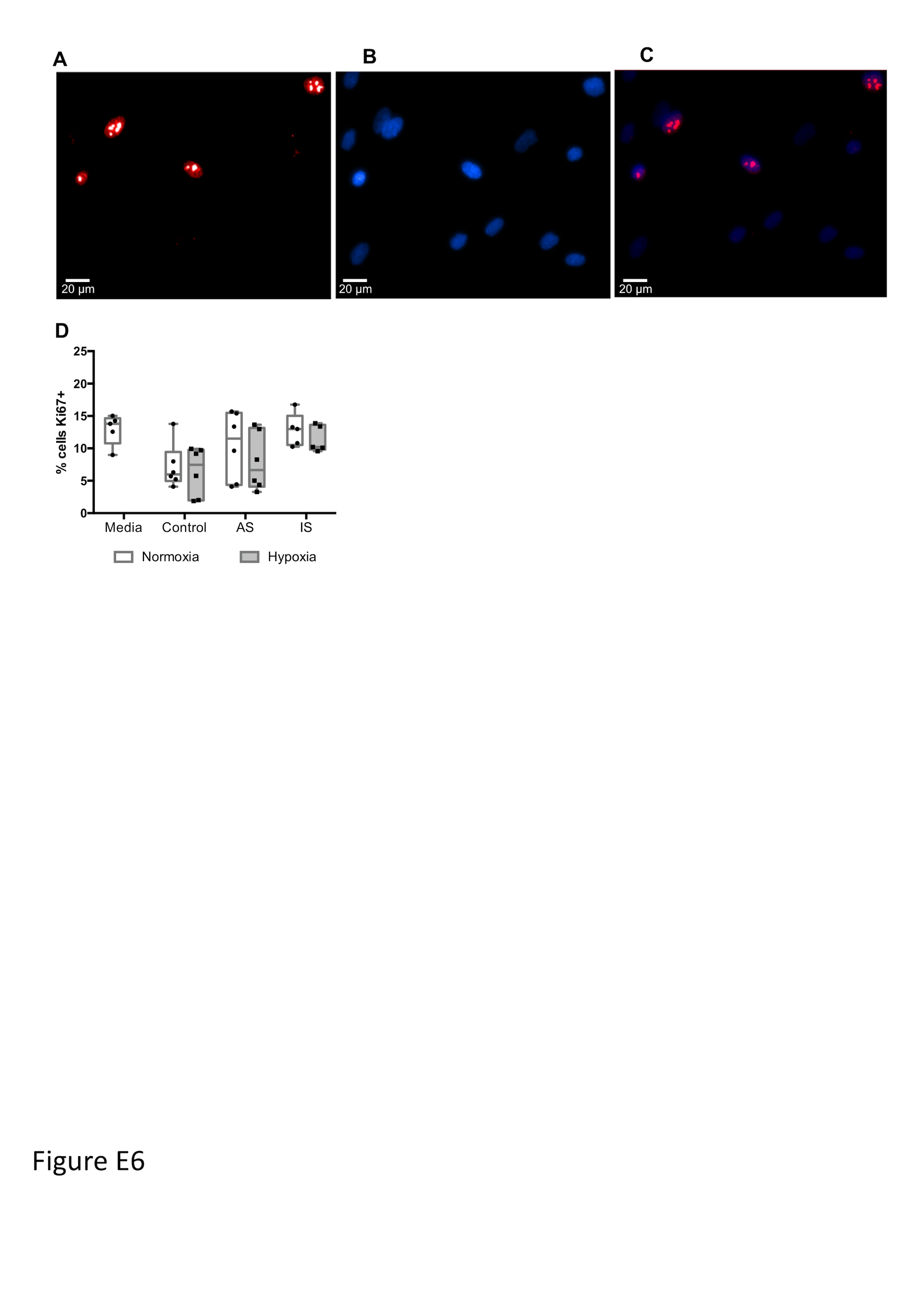

Supplement: Supplementary file 7 [file Image_6.tiff]

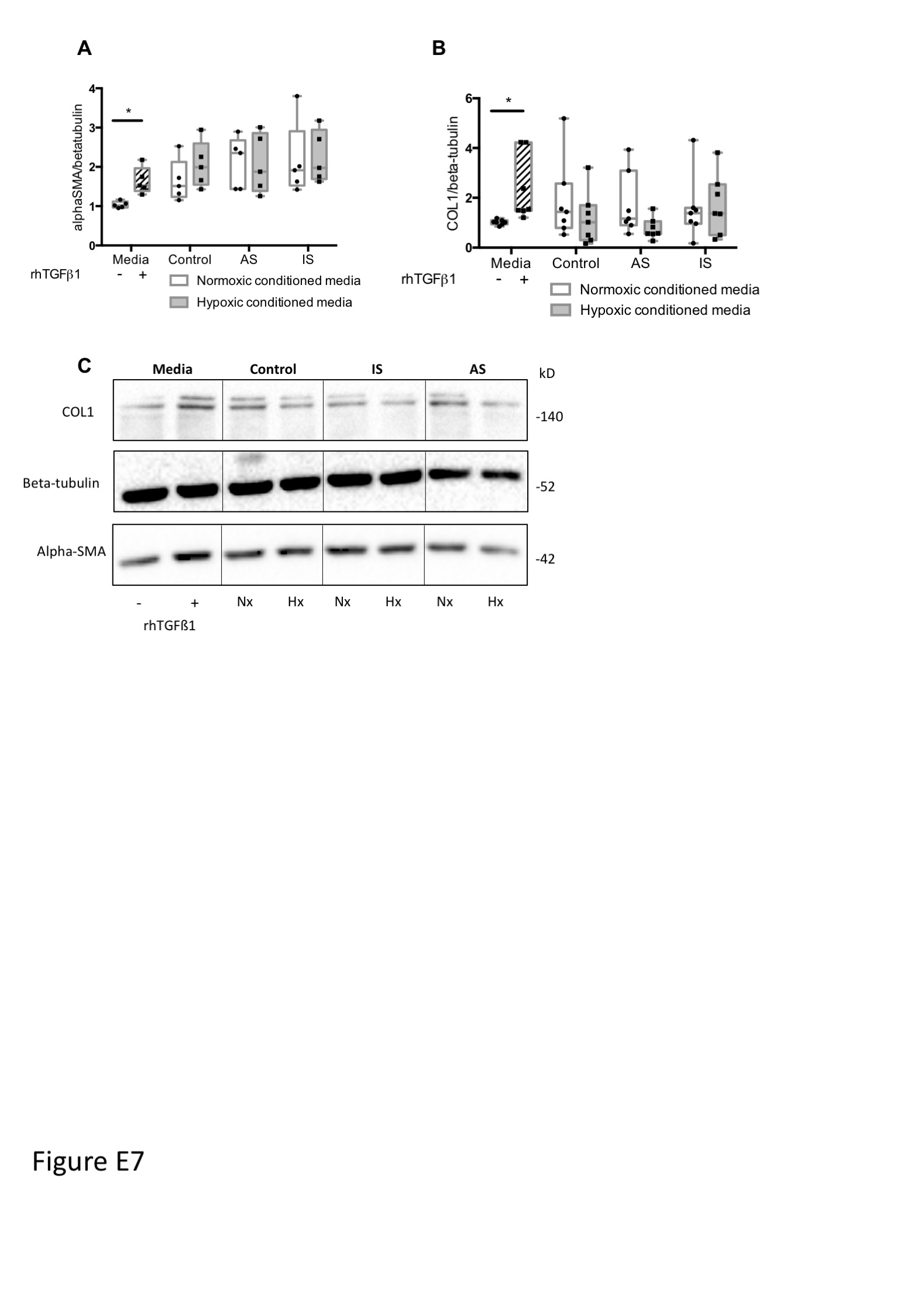

Supplement: Supplementary file 8 [file Image_7.jpeg]
